# Direct Assessment of Wall Shear Stress by Signal Intensity Gradient from Time-of-Flight Magnetic Resonance Angiography

**DOI:** 10.1155/2017/7087086

**Published:** 2017-08-16

**Authors:** Kap-Soo Han, Sang Hyuk Lee, Han Uk Ryu, Se-Hyoung Park, Gyung-Ho Chung, Young I. Cho, Seul-Ki Jeong

**Affiliations:** ^1^Research Institute of Clinical Medicine, Chonbuk National University, Biomedical Research Institute of Chonbuk National University Hospital, Jeonju, Jeonbuk 54907, Republic of Korea; ^2^Equipment Qualification Center for Nuclear Power Plants, Korea Institute of Machinery and Materials, Daejeon 34103, Republic of Korea; ^3^Department of Neurology, Chonbuk National University Medical School and Hospital, Jeonju, Jeonbuk 54907, Republic of Korea; ^4^Pixoneer Geomatics, Inc., Daejeon 34126, Republic of Korea; ^5^Department of Radiology, Chonbuk National University Medical School and Hospital, Jeonju, Jeonbuk 54907, Republic of Korea; ^6^Department of Mechanical Engineering and Mechanics, Drexel University, Philadelphia, PA 19104, USA

## Abstract

The aim of the study was to calculate the arterial wall signal intensity gradient (SIG) from time-of-flight MR angiography (TOF-MRA) and represent arterial wall shear stress. We developed a new algorithm that uses signal intensity (SI) of a TOF-MRA to directly calculate the signal intensity gradient (SIG). The results from our phantom study showed that the TOF-MRA SIG could be used to distinguish the magnitude of blood flow rate as high (mean SIG ± SD, 2.2 ± 0.4 SI/mm for 12.5 ± 2.3 L/min) and low (0.9 ± 0.3 SI/mm for 8.5 ± 2.6 L/min) in vessels (*p* < 0.001). Additionally, we found that the TOF-MRA SIG values were highly correlated with various flow rates (*β* = 0.96, *p* < 0.001). Remarkably, the correlation coefficient between the WSS obtained from the computational fluid dynamics (CFD) analysis and the TOF-MRA SIG was greater than 0.8 in each section at the carotid artery (*p* < 0.001 for all *β* values). This new technique using TOF-MRA could enable the rapid calculation of the TOF-MRA SIG and thereby the WSS. Thus, the TOF-MRA SIG can provide clinicians with an accurate and efficient screening method for making rapid decisions on the risk of vascular disease for a patient in clinical practice.

## 1. Introduction

The hemodynamic parameters of blood flow, such as blood pressure and arterial wall shear stress (WSS), provide important information about the pathophysiological mechanisms underlying vascular diseases [1]. Blood pressure, the normal stress acting against the arterial wall, is relatively easy to measure and has been widely used as a biomarker for vascular diseases [1]. Arterial WSS, the stress tangential to the arterial wall, also has been reported to play a pathophysiological role in endothelial function [2] and arterial thromboembolism [3]. However, there is substantial technical difficulty in estimating WSS in clinical practice.

Several techniques are used to estimate arterial WSS, including ultrasonography, computational fluid dynamics (CFD), and phase-contrast magnetic resonance (MR). The ultrasonographic method is used to measure the flow velocity and arterial diameter and provides average values for the shear stress over the entire target lumen [4]. CFD overcomes the limitations of the ultrasonographic method [5] and provides vital information on blood flow and wall parameters, rendering them with realistic arterial geometries [6]. However, CFD results are largely dependent on defined simulation conditions, such as arterial geometries, flow velocity profiles, arterial wall properties, and blood characteristics [7]. Phase-contrast MR provides direct information on the flow velocity [8], but previous studies suggested that it was not appropriate for estimating arterial WSS [9]. The usefulness of estimating WSS using phase-contrast MR imaging in clinical practice remains limited because of the unknown relevance of WSS and questions regarding its accuracy due to low spatial resolution with respect to the boundary layer along the arterial wall. However, a rapid assessment of the WSS is crucial for screening patients with vascular disorders and deciding whether or not further examination is needed.

Current TOF-MRA techniques to control intraluminal saturation are universally applied to every subject and are not individually customized. If intraluminal saturation is variable according to anatomical regions and subjects, it might be caused by the individual characteristics of the arterial geometry and/or flow velocity but not by the TOF-MRA techniques. Moreover, we can assume that the signal intensity along the arterial wall could provide information unique to each subject. For the diagnosis of arterial geometry, arterial signal intensity should be more accurate at the periphery (near the wall) than in the central region. Our hypothesis was that the arterial wall signal intensity gradient (SIG) from TOF-MRA can be calculated to represent arterial wall shear stress. Thus, the objective of the present study was (1) to develop a unique method to calculate the TOF-MRA arterial wall SIG, (2) to perform validation studies to determine if the TOF-MRA SIG is flow rate and geometry dependent, and (3) to compare the results with wall shear stress values obtained from computational fluid dynamics (CFD).

## 2. Material and Methods

**Figure 1:**
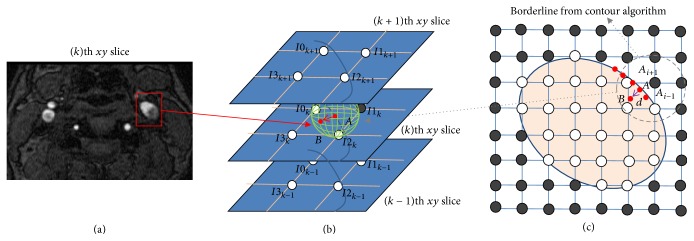
Calculation of TOF-MRA signal intensity gradient (SIG). (a) TOF-MRA axial source image. (b) 3-dimensional gradient vector (red arrow) of the maximum intensity change. (c) Positioning of points (*A* and *B*): point *A* is a reference point on the artery wall (contour line) and point *B* is 0.03 mm distant from point *A*.

**Figure 2:**
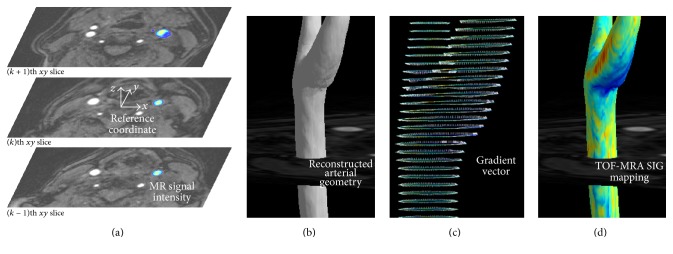
Visualization and TOF-MRA signal intensity gradient (SIG). (a) The reference coordinate settings on TOF-MRA axial source images and the selected (left carotid) arterial color display. (b) 3D reconstruction of arterial geometry using the arterial threshold value. (c) A gradient vector setting: from the reference point on the artery wall (contour line), the position of inner point is identified with both a specific distance (0.03 mm in the present study) and the direction having the maximum gradient of the signal intensity. For the drawing, the gradient vector is lengthened to 0.3 mm. (d) 3D mapping of the carotid arterial TOF-MRA SIG.

In this section, we describe a new method to calculate the SIG from TOF-MRA image, reconstruct the arterial, and map the calculated SIG on the arterial model. Figures 1 and 2 depict an overview of algorithm and our image processing steps, which are further detailed in Sections 2.1 and 2.2. The sequences and parameters of the current TOF-MRA are described in Sections 2.3.1 and 2.3.2 in detail. An abstract was presented in XXII World Congress of Neurology (WCN 2015) in Santiago, Chile [10].

### 2.1. A Method to Calculate the Arterial Wall SIG Using TOF-MRA

#### 2.1.1. Calculation of Signal Intensity Gradient (SIG)

Axial TOF-MRA source image of the carotid artery was obtained, and the arterial region was characterized for the SIG calculation (Figure 1(a)). The SIs at the isopoint (Φ_*a*_) and at the inner point (Φ_*b*_) were calculated by using a trilinear interpolation algorithm based on the positions and SIs in the eight neighboring voxels (Figures 1(b) and 1(c)). For each isopoint (point *A*), the SIG was calculated from the difference in SI between points *A* and *B* as follows:

(1) Scalar  SIG,  SI/mm=Φb−ΦaXb−Xa,Vector  SIG,  SI/mm=Φb−ΦanXb−Xa.

#### 2.1.2. Reference Coordinate Setting

The rectangular coordinate is defined with the spatial information including the TOF-MRA. For the coordinate, the position for the center of TOF-MRA volumetric data is** X**_0,0,0_ = (0, 0, 0), and the 3D voxel structure (*i*,* j*,* k*, spatial index) is constructed with the intervals for each axis (*dx*,* dy*,* dz*, in mm units as specified in TOF-MRA) as **X**_*i*,*j*,*k*_ = (*dx* · *i*, *dy* · *j*, *dz* · *k*), as shown in Figure 2(a).

#### 2.1.3. Arterial Wall Setting and 3D Reconstruction of Arterial Geometry

Isopoints having specific values of signal intensity are identified in each image of the TOF-MRA. The position (**X**_*a*_) of each isopoint (point *A*, for arterial contour line) was defined as** X**_*a*_ = (*x*_*a*_,* y*_*a*_,* z*_*a*_). For the 3D arterial geometry, the isosurface was modeled with isolines for each image of the TOF-MRA, as shown in Figure 2(b).

#### 2.1.4. Visualization of Signal Intensity Gradient (SIG)

For each isopoint (point *A*), the position (**X**_*b*_) of the inner point (point *B*) was identified with a specific distance* d* of 0.03 mm in the present study and the direction having the maximum gradient of SI (**n**) as follows (Figure 2(c)):

(2) Xb=Xa+dn,n=∇Φ∇Φ.

With the 3D reconstructed arterial geometry, the SIGs at the isopoints were depicted as shown in Figure 2(d).

### 2.2. Normalization of MR Signal Intensity

For each voxel, the MR signal intensity (*Φ*_*i,j,k*_) is recorded with the normalization process of raw data (*φ*_*i,j,k*_) as follows:

(3) Φi,j,k=φi,j,k−μφσφSI,  signal  intensity,  as  arbitrary  unit,

where *μ*_*φ*_ denotes the mean value and *σ*_*φ*_ is the standard deviation of the MRA dataset. The normalization was based on the assumption that the imaging regions, such as the brain or neck, have similar statistical distributions (e.g., histogram shapes) across different MR machines. The normalization process could eliminate the offset (mean) and scale (standard deviation) effect across different datasets while maintaining the statistical distribution.

### 2.3. Validation of the TOF-MRA SIG

In this section, we conducted a series of validation experiments such as a phantom study and the preclinical trial study using carotid arteries obtained from healthy subjects. The new algorithm used the raw signals from TOF-MRA, which was based on flow-related enhancement caused by the inflow of fully magnetized spins into the imaging volume [11]. Since the vascular signal in SIG essentially depends on the flow velocity [m/s], SIG is conceptually correspondent to shear rate [s^−1^]. Since WSS has the unit of Pascal, SIG can conceptually have the unit of WSS if blood viscosity [Pa·s] is multiplied to SIG.

#### 2.3.1. Phantom Study for the Flow Rate Dependency

**Figure 3:**
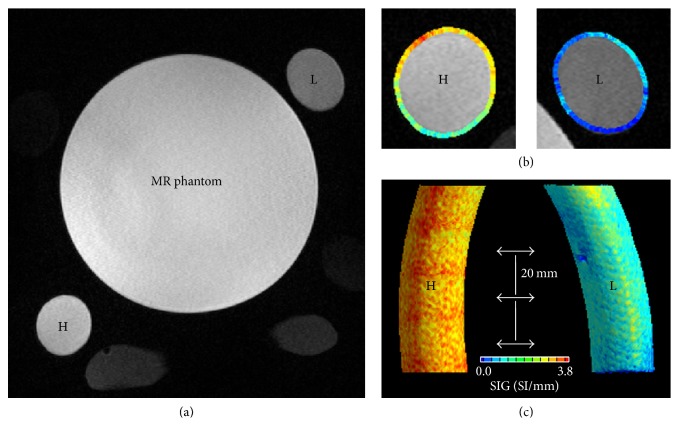
A phantom experiment: flow-rate dependency of TOF-MRA SIG. (a) Two tubes (80 mm length) of high flow rate (H, 12.5 ± 2.3 L/min of mean flow rate) and low flow rate (L, 8.5 ± 2.6 L/min). In the middle, a MR phantom was observed. (b) Axial view of TOF-MRA SIG in the H and L tubes. (c) 3D reconstructed tubes depicting TOF-MRA SIG: TOF-MRA SIG values were significantly higher in the H tube (mean ± SD, 2.2 ± 0.4, SI/mm) than in the L tube (0.9 ± 0.3, *p* < 0.001). TOF-MRA SIG values were measured at three levels at 20 mm intervals.

A phantom study investigating the TOF-MRA SIG as a function of flow rate was performed and the results are shown in Figure 3. Two flow phantoms with different flow rates (H tube for high flow versus L tube for low flow) were constructed from flexible tubing (inner diameter 2.1 cm). Tap water (T1 time, 2.1–3.5 s) was continuously pumped and passed through the tubes and the imaging region of a 3.0-T MR imaging system (Verio 3.0 T, Siemens, Germany). The MR phantom, a 1900 mL plastic bottle containing 3.75 g NiSO_4_  *∗* 6H_2_ + 5 g NaCl per 1000 mL of sterilized H_2_O (T1, 40–120 ms), was placed between the tubes. The imaging parameters for the 3-dimensional (3D) TOF-MRA in the phantom study were as follows: repetition time (TR)/echo time (TE) = 22.0/3.6 ms; flip angle = 18.0°; field of view (FOV) = 190 × 210 mm; matrix size = 384 × 296; sensitivity encoding (SENSE) factor = 2.5; slice thickness = 0.5 mm; and number of averages (NEX) = 1. The TOF-MRA SIG was calculated semiautomatically with software developed in-house using both 2-dimensional source images and 3D reconstructed formats.

#### 2.3.2. 3D TOF-MRA and CFD of the Human Carotid Artery

**Figure 4:**
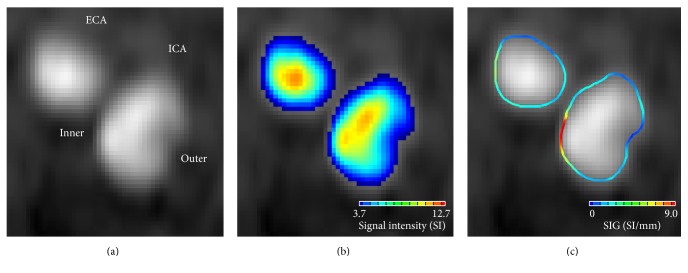
TOF-MRA axial source image for the right internal carotid (ICA) and external carotid artery (ECA). (a) At the arterial periphery, the signal was darker near the outer wall of ICA than near the inner wall because of the difference in intraluminal saturation. (b) Color displays of the arterial signal intensity showed a clear gradation in the color scale from the arterial center region to the periphery. (c) TOF-MRA SIG showed the regions with high or low SIG values.

Extracranial carotid artery 3D TOF-MRA was performed on five healthy volunteers for a comparative study at Chonbuk National University Hospital. The imaging parameters for the 3D TOF-MRA scan were as follows: TR/TE = 23/3.5 ms; flip angle = 20.0°; FOV = 200 × 200 mm; matrix size = 488 × 249; SENSE factor = 2.5; slice thickness = 1.0 mm; and NEX = 1. The TOF-MRA scan duration was 4 min on a 3.0-T MR system (Achieva 3.0 T, Philips, Netherlands). To obtain the TOF-MRA SIG values of the carotid artery, the threshold values were set as low as possible to include all arterial signal intensities with postmorphological noise exception processes, as shown in Figure 4. The time required to calculate the TOF-MRA SIG for each subject was less than 2 min using a laptop running a Windows OS with a 3.6 GHz Intel Core i7 64-bit processor.

The arterial WSS was calculated from the blood flow simulation using a commercial CFD program (STAR-CD version 4.14, CD-Adapco, London, UK). For the carotid arterial model, a rigid arterial wall without compliance was assumed, and the computational domain was modeled with MRA source images using the same threshold values as those used for the TOF-MRA SIG. For each carotid artery, steady blood flow simulations were performed with three different flow conditions: peak systolic, end diastolic, and mean. The flow velocities in the common carotid artery, internal carotid artery, and external carotid artery were measured from ultrasonographic examinations, as described previously [12]. Regarding the blood properties, the density and dynamic viscosity were assumed to be 1,004 kg/m^3^ and 3.5 cP, respectively. Detailed information regarding the blood flow simulation in the carotid artery was explained in a previous study [13]. Under these simulation conditions, it took approximately 2 hours using a Linux cluster with four CPUs each with a 2.5 GHz AMD Opteron 64-bit processor.

Finally, for each carotid artery, the TOF-MRA SIG and the CFD WSS were obtained at six different levels from the CCA (i.e., level 1) to the carotid bulb (i.e., level 6) at 2.5-mm interval. At each level, both the arterial SIG and WSS were obtained at 12 sites at 30° interval from the equatorial line of the left lateral axis. Overall, 12 arterial sections at six different levels from five carotid arteries were analyzed.

Independent* t*-tests were used to assess differences in the continuous variables, and Pearson's correlation was used to obtain coefficients. Statistical analyses were conducted using SPSS version 20 (SPSS, Chicago, IL, USA).

## 3. Results

### 3.1. Geometry Dependency of the TOF-MRA SIG

Remarkably, the TOF-MRA SIG could be differentiated based on the carotid arterial geometry; see Figure 4. At the arterial periphery (seen in the TOF-MRA carotid axial source image), the signal was darker near the outer wall of the internal carotid artery (i.e., the low-shear zone) than near the inner wall (i.e., the high-shear zone), because of the difference in intraluminal saturation; see Figures 4(a) and 4(b). Thus, the regional difference in signal loss enabled the SIG to differentiate between the high- and low-shear zones; see Figure 4(c).

### 3.2. Flow Rate Dependency of the TOF-MRA SIG

The TOF-MRA SIG was calculated semiautomatically with a software developed in-house using 2D source images. We applied two different mean flow rates (high [H tube] = 12.5 ± 2.3 L/min and low [L tube] = 8.5 ± 2.6 L/min) and performed the SIG analysis. The TOF-MRA SIG values were significantly higher in the H tube (mean ± SD = 2.2 ± 0.4, SI/mm) than in the L tube (mean ± SD = 0.9 ± 0.3, *p* < 0.001). The calculated TOF-MRA SIG values significantly correlated with variable flow rates (*β* = 0.96, *p* < 0.001). Our analysis with the phantom study clearly showed that the TOF-MRA SIG was dependent on the flow rate.

### 3.3. Comparison of TOF-MRA SIG and WSS of CFD

**Figure 5:**
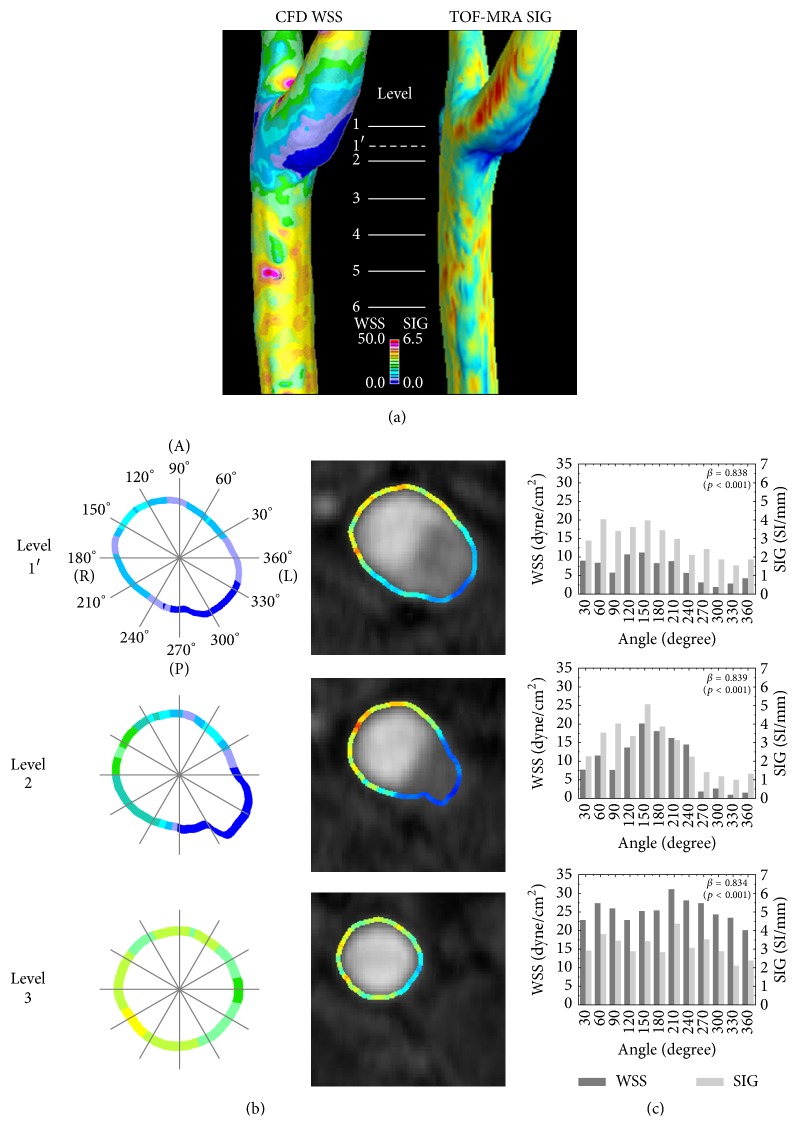
3D (a) and cross-sectional (b) views of wall shear stress (WSS) from computational fluid dynamics (CFD) and TOF-MRA SIG in carotid artery. A comparison (c) of TOF-MRA SIG with wall shear stress from CFD. Labels indicate the levels where CFD WSS and TOF-MRA SIG were obtained and compared. In each section, TOF-MRA SIG values were significantly correlated with CFD WSS (all *β* > 0.8, *p* < 0.001).

Because the new algorithm for the TOF-MRA SIG depended on the flow rate and the arterial geometry, the final step was to investigate whether the TOF-MRA SIG represented the arterial WSS. We estimated the WSS of the carotid artery using CFD and compared the results with those from the TOF-MRA SIG, as shown in Figure 5(a). Note that cross-sectional figures and correlation values are presented in Figure 5(b). At each level, the WSS values measured by CFD and TOF-MRA SIG had correlation coefficients of greater than 0.8 (*p* < 0.001 for all values, see Figure 5(c)). In summary, the TOF-MRA SIG tends to be dependent on both flow rate and arterial geometry and is highly correlated with the WSS determined by CFD.

## 4. Discussion

TOF-MRA is based on the flow-related enhancement from the inflow of fully magnetized spins into the imaging volume. For typical flow profiles where the velocity is not uniform across the diameter (i.e., the velocity is the highest in the center of the lumen and the lowest near the wall), the flow-related enhancement is also nonuniform. Although some previous studies investigated arterial flow velocity by tracking presaturated or excited spins [14–17], TOF-MRA has been developed for its improved spatial resolution of arterial geometry, which reaches submillimeter levels [18]. In terms of TOF phenomena, some features (e.g., hemodynamic data) should be compromised in favor of others (e.g., geometry) because of the parabolic distribution of blood flow, such as washout versus the phase shifts of excited spins caused by motion along the magnetic field gradients [11]. Signal intensities in the central arterial region are susceptible to washout of excited spins due to the highest flow velocity, while those at the arterial periphery are prone to intraluminal saturation due to the lowest flow velocity [19].

For the diagnosis of the arterial geometry, the arterial SI should be more preserved at the periphery (which is prone to intraluminal saturation) than in the central region (which is susceptible to washout of excited spins). Current TOF-MRA gradient echo sequence, including short repetition time, partial flip angle, multiple thin-slab acquisition [20], and tilted optimized nonsaturating excitation pulses [21], is effective in reducing the intraluminal saturation. The TOF-MRA techniques are universally applicable to all patients. Since the signal intensity from the TOF-MRA techniques depends on the hemodynamic characteristics, the arterial signal intensity according to the degrees of intraluminal saturation is subject to the individual characteristics such as arterial geometry and flow velocity [18, 19]. Therefore, the TOF-MRA SIG, which represents the gradient vector from the arterial contour line of signal intensity, is a physiological in vivo representation on arterial hemodynamics.

We used CFD as a computational method for representing WSS [22]. Although the correlation coefficients were robustly high between CFD WSS and TOF-MRA SIG, data inversion did occur. For example, at the carotid bulb, the CFD WSS was lower than the TOF-MRA SIG values, whereas the CFD results were higher in the CCA segments. It might have been caused by the calculation methods for the CFD WSS and the TOF-MRA SIG. The TOF-MRA SIG showed the relative difference of SI along the arterial contour, whereas the CFD calculated WSS utilizing Newtonian blood viscosity of 3.5 cP and shear rates based on the geometric 3D substrate. Accordingly, at levels 1 and 2, where the corresponding shear rates were known to be relatively small due to bifurcation-induced flow recirculation compared to the shear rate at level 3, the CFD WSS might have been underestimated, while the TOF-MRA SIG have been overestimated. Further studies are needed to clarify how the geometric reformat, boundary setting, and the use of patient-specific non-Newtonian blood viscosity affect WSS calculation.

We did not perform a comparative study between the TOF-MRA SIG and other methods for determining WSS, such as phase-contrast MR. Phase-contrast MR, which is based on phase display, can provide directional flow information because it encodes variable velocities [23, 24]. Although new sequences for phase-contrast MR have been developed and more useful methods are anticipated [25], its use remains quite limited in current clinical practice [26]. Therefore, a study comparing the WSS obtained from phase-contrast MR with that from TOF-MRA SIG would be of significant interest in future studies.

Limitations of the present study include the following: first, the present study calculated the TOF-MRA SIG in arteries where the measurement plane (*x*-*y* plane) was perpendicular to the flow direction, such as in the carotid artery. Subsequent studies are needed to define whether the TOF-MRA SIG could be utilized in other arteries with various flow axes, such as middle cerebral artery. Second, the arterial wall in the CFD model was assumed to be rigid. Future study with a fluid structure interaction (FSI) model for the compliant arterial wall and non-Newtonian viscosity modeling considering the shear-thinning effect of blood is needed to define their associations better. Third, we did not perform a comparative study between the TOF-MRA SIG and other methods to determine WSS, such as phase-contrast MR. Last, the present study used a normalization process for the TOF-MRA SIG in the human carotid artery. The normalization process still lacks direct evidence regarding whether it can eliminate both offset and scale effects across different MRA datasets. For this issue, we are preparing a subsequent study to support the usefulness of the normalization process in TOF-MRA in humans. Separately, further studies with the TOF-MRA SIG will be performed in a large group of healthy subjects and patients to obtain the robustness of the findings and the sensitivity of the technique to identify regional changes of arterial WSS.

## 5. Conclusion

We developed a novel algorithm for calculating the TOF-MRA SIG, which was dependent on both flow rates and arterial geometry. We showed that it is possible to rapidly assess the WSS by using the TOF-MRA SIG. Also, we demonstrated the significant correlations between the TOF-MRA SIG and the CFD WSS. Thus, we anticipate that the SIG information from the TOF-MRA can further provide readily available guidance on arterial health, pathologies, and long-term vascular outcomes.
